# Editorial: Siblinghood through any disability: the state of the art and future directions

**DOI:** 10.3389/fpsyg.2025.1670389

**Published:** 2025-08-21

**Authors:** Annalisa Levante, Serena Petrocchi, Flavia Lecciso

**Affiliations:** ^1^Department of Human and Social Sciences, University of Salento, Lecce, Italy; ^2^Lab of Applied Psychology, Department of Human and Social Sciences, University of Salento, Lecce, Italy; ^3^Faculty of Biomedical Sciences, Institute of Family Medicine, Università della Svizzera Italiana, Lugano, Switzerland

**Keywords:** sibling, siblinghood, resolution of the diagnosis, sibling relationships, family interaction, family dynamics, interpersonal adjustment, psychological wellbeing

This Editorial aims to summarize the key themes addressed by the articles published in the Research Topic entitled “*Siblinghood through any disability: the state of the art and future directions*,” which explores the experiences of siblings of individuals with disabilities. Over the past few years, scholars have devoted increasing attention to the roles served and the experiences lived by siblings of children with disabilities. Recent systematic reviews and empirical studies (e.g., Cervellione et al., 2025; Lecciso et al., 2025; Levante et al., 2024; Lee and Shikarpurya, 2025; Levante et al., 2023; Alon, 2025) have highlighted that the main topics explored include sibling relationships, the roles taken on in supporting their brother or sister with a disability, and the siblings' social and personal functioning. The articles included in this Research Topic address many of the themes previously identified in the literature while also introducing new perspectives that contribute to shaping future research directions.

This Research Topic includes a total of seven contributions from five countries: Italy (*n* = 2), Norway (*n* = 2), Poland (*n* = 1), Israel (*n* = 1), and the United Kingdom (*n* = 1). Altogether, the articles involved 38 authors and 14 expert reviewers in the fields of family systems dynamics and siblinghood. Of the included papers, four adopted a quantitative design, two employed a qualitative approach, and one utilized a mixed-methods design. The quantitative studies primarily focused on family dynamics involving siblings and parents within the context of various disabilities. Both risk and protective factors influencing sibling adjustment and family interaction have been identified. For instance, Łada-Maśko et al. delineated two distinct family interaction profiles: strained vs. resilient. The former are characterized by low cohesion and communication, hyper-protection, and inconsistent parenting, while resilient families exhibit positive interaction dimensions. Although the authors found that the strained profile was the most prevalent, all families fell into both profiles regardless of the presence or absence of disability. In other words, dysfunctional family interactions are not exclusive to families with a child with disability.

Lecciso et al. tested the protective role of parental resolution of the child's diagnosis in facilitating siblings' acceptance and fostering closer and less conflictual sibling relationships. Specifically, parental resolution is a predictor of sibling resolution of the diagnosis, which, in turn, serves as a protective factor for a high-quality sibling relationship. Rum et al. highlighted the beneficial impact of having older typically developing siblings on the social functioning of autistic children, emphasizing the role of siblings as valuable resources for social development, regardless of the older sibling's neurodiversity status. Finally, the longitudinal study by Zahl et al. explored bidirectional influences between parental and sibling mental health and adjustment over time. The findings revealed no significant cross-domain longitudinal associations, neither between sibling mental health and adjustment nor between parental and sibling outcomes. Across all models, only self-feedback effects within each domain reached significance. No associations were found between changes in maternal and sibling mental health; however, a small but significant correlation emerged between changes in maternal mental health and sibling adjustment.

Complementing these results, two qualitative studies provided in-depth insights into siblings' knowledge, perceptions, and emotional experiences related to living with a brother/sister with a disability. Fjermestad and Lervik revealed significant gaps in siblings' understanding of autism, particularly among young children, and highlighted the potential for targeted educational interventions in promoting awareness and reducing misconceptions. Foley et al. investigated the lived experiences of typically developing twins and their mothers in the context of neurodivergence, identifying relational challenges, perceived difficulties, and unmet support needs. The findings suggested that post-diagnostic support targeting communication strategies and emotional guidance could substantially enhance sibling relationships and overall family dynamics.

The mixed-methods study by Battistin et al. investigated the quality of the sibling relationship and the role of social play among visually impaired and sighted siblings. By combining quantitative and qualitative methodologies, the authors demonstrated that sibling relationships, where one sibling has a diagnosis, can be harmonious and not necessarily marked by difficulty. Social play emerged as a key protective factor, fostering strong sibling bonds and supporting their mental health.

Taken altogether, the contributions offer a multidimensional perspective on the complex influences shaping siblings' adjustment and family dynamics in the context of disability. They underscore the importance of integrating both empirical data and personal narratives to fully capture the sibling experience. Importantly, the findings of the research highlighted the need for systemic and family-centered interventions that consider not only the emotional and social needs of parents (Lecciso et al., 2016, 2013; Sher-Censor and Shahar-Lahav, 2022) and individuals with disabilities (De Carlo et al., 2024; Martis et al., 2024) but also those of siblings.

Future directions for research include longitudinal studies that investigate sibling and family adjustment across different developmental stages to better understand the nature of these relationships. Moreover, there is a need to explore diverse disability diagnoses and cultural contexts to enhance the generalizability of results. Research should also examine the effectiveness of targeted interventions designed to support siblings' coping and resilience. Finally, incorporating paternal perspectives and expanding the methodology to include mixed-method approaches are key steps toward a more holistic understanding of family dynamics, enabling more targeted and meaningful support for families living with disability.
